# One-Year Follow-Up of a Series of 100 Patients Treated for Lumbar Spinal Canal Stenosis by Means of HeliFix Interspinous Process Decompression Device

**DOI:** 10.1155/2014/176936

**Published:** 2014-04-15

**Authors:** Alberto Alexandre, Andrea Maria Alexandre, Mario De Pretto, Luca Corò, Raul Saggini

**Affiliations:** ^1^European Neurosurgical Institute (EUNI), Via Chira 2, 31100 Treviso, Italy; ^2^Istituto di Radiologia, Università Cattolica, Largo A. Gemelli, 00100 Rome, Italy; ^3^Istituto di Fisiatria, Università di Chieti, Via dei Vestini 31, 66100 Chieti, Italy

## Abstract

*Purpose.* New interspinous process decompression devices (IPDs) provide an alternative to conservative treatment and decompressive surgery for patients with neurogenic intermittent claudication (NIC) due to degenerative lumbar spinal stenosis (DLSS). HeliFix is a minimally invasive IPD that can be implanted percutaneously. This is a preliminary evaluation of safety and effectiveness of this IPD up to 12 months after implantation. *Methods.* After percutaneous implantation in 100 patients with NIC due to DLSS, data on symptoms, quality of life, pain, and use of pain medication were obtained for up to 12 months. *Results.* Early symptoms and physical function improvements were maintained for up to 12 months. Leg, buttock/groin, and back pain were eased throughout, and the use and strength of related pain medication were reduced. Devices were removed from 2% of patients due to lack of effectiveness. *Conclusions.* Overall, in a period of up to 12-month follow-up, the safety and effectiveness of the HeliFix offered a minimally invasive option for the relief of NIC complaints in a high proportion of patients. Further studies are undertaken in order to provide insight on outcomes and effectiveness compared to other decompression methods and to develop guidance on optimal patient selection.

## 1. Introduction

Degenerative lumbar spinal stenosis (DLSS) is the most common type of spinal stenosis, with a reported incidence of 2–8% in the general population [1]. Due to the increase in life duration DLSS condition becomes symptomatic and is one of the most common causes for spinal surgery in the elderly population over the age of 50. Neurogenic intermittent claudication (NIC) is the cardinal symptom of DLSS, presenting as numbness, weakness, and discomfort in the legs (with or without back pain) during walking or prolonged standing. One of the characteristic findings in NIC is the regression of symptoms when sitting or forward bending [2]. Lumbar extension decreases the cross-sectional area of the dural sac as well as the neural foramina, causing compression on neural and vascular structures, which triggers exacerbation of symptoms [3–5].

Treatment of LSS begins with nonsurgical medical management [6–9]. While some patients respond well to this treatment, many do not and consequently they become candidates for surgical intervention. Decompressive lumbar laminectomy is the most common surgical procedure, with or without fusion in consideration of coexistence of spinal instability [10–30].

Interspinous process decompression (IPD) is a minimally invasive spinal surgery in which an implant is placed between the adjacent spinal processes of the symptomatic disc level. The intraspinal process decompression system was developed for patients who have LSS with disabling neurogenic intermittent claudication and who are able to relieve their symptoms when they bend forward or flex their spine. The IPD is designed [31–38] to limit pathological extension of the spinal segments and to maintain them in a neutral or slightly flexed position which may allow patients to resume their normal posture rather than to flex the entire spine to gain symptomatic relief.

Interspinous process decompression devices (IPDs) provide an alternative therapy to conservative treatment and are less invasive than open decompressive surgery for patients suffering from NIC. Biomechanical studies have shown that IPDs significantly reduce intradiscal pressure and facet load and prevent narrowing of the spinal canal and neural foramen [39].

HeliFix device is an IPD that can be implanted percutaneously. It is indicated for the treatment of patients suffering from 1- or 2-level symptomatic DLSS (from L1 to L5) with NIC, exacerbated in the upright posture or by extension of the lumbar spine and relieved by flexion. The procedure has the great advantage of preserving muscular integrity, thereby respecting neuromuscular system.

We present a study of 100 consecutive eligible cases, to assess safety and effectiveness of this IPD up to 12 months after procedure.

## 2. Patients and Methods

### 2.1. Patients

HeliFix IPD was implanted at symptomatic levels in 100 patients, 42 men and 58 women, of mean age 56 years (range 32–81 years). All patients were selected on the basis of a history of DLSS (from L1 to L5, L5-S1 level is not considered for this device), confirmed by MRI, with symptoms of NIC, including leg/buttock/groin pain, with or without back pain, relieved by flexion. If back pain was also present, it was to be partially relieved when flexed. Patients had to be able to sit for 50 min without pain and walk a distance of 20 m without pain.

NIC was observed preoperatively in 84 patients, low back pain in 62, and leg pain in 47.

Mean VAS was 8, and mean Roland Morris score was 18.

Disability, which is defined after standing laterolateral X-rays, was present in 74 patients as angular instability (ROM > 10°) and/or 1st degree (<4 mm) sagittal degenerative spondylolisthesis. Data are summed in Table 1.

### 2.2. Surgical Technique

All patients have been treated in the prone position (lying flat, face downwards): 3% under general anesthesia and 97% receiving a local anesthetic and mild sedation. After radiographic identification of the surgical level, a 1.5 cm incision is made at the affected level, 6–10 cm lateral to the midline.

The IPD is inserted in the interspinous space after preparing the space by opening the interspinous ligament and after determining the shape of the device. These two targets are reached by using a series of proof elements which are collocated on the tip of trocars.

**Figure 1:**
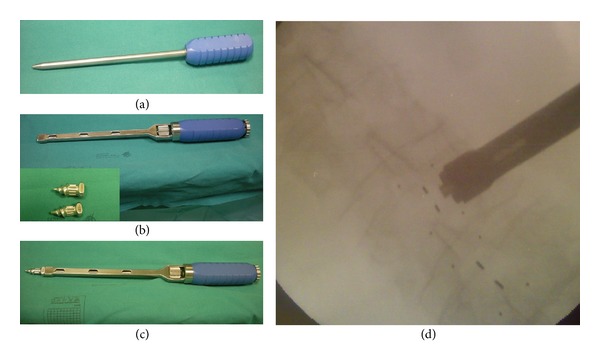
The first sharp inserted in the interspinous space is a 5 mm thick trocar (a). After his removal a second trocar bringing on the tip a proof element of 8 mm size is introduced ((b) and (c)). The percutaneous insertion of the IPD is controlled under fluoroscopy (d).

Under fluoroscopy, the first sharp, 5 mm thick, trocar is introduced and advanced through the interspinous space. After its removal another trocar bringing on the tip a proof element of 8 mm size is introduced. The percutaneous insertion of increasing size proof elements of 10, 12, 14, and 16 mm allows a gradual distraction of the interspinous area to measure the optimal decompression and prevent overdistraction (Figure 1).

The size of the needed IPD is determined by monitoring under lateral fluoroscopic X-rays the dilation of the disc height, comparing it with the disc height observed at the beginning of the procedure. Simultaneously the other criterion for determining the size of the needed IPD is the resistance which is met in screwing the sharp proof elements in the interspinous pace. A rather hard resistance needs to be met, as well as the sensation of bone grasping.

Once the size is determined, the definitive HeliFix device is secured on the trocar and introduced. The IPD device follows an innovative concept that features an implant with a self-distracting helical tip which is “screwed” in the interspinous space, working with the intact supraspinous ligaments to keep it in place.

### 2.3. Follow-Up

**Figure 2:**
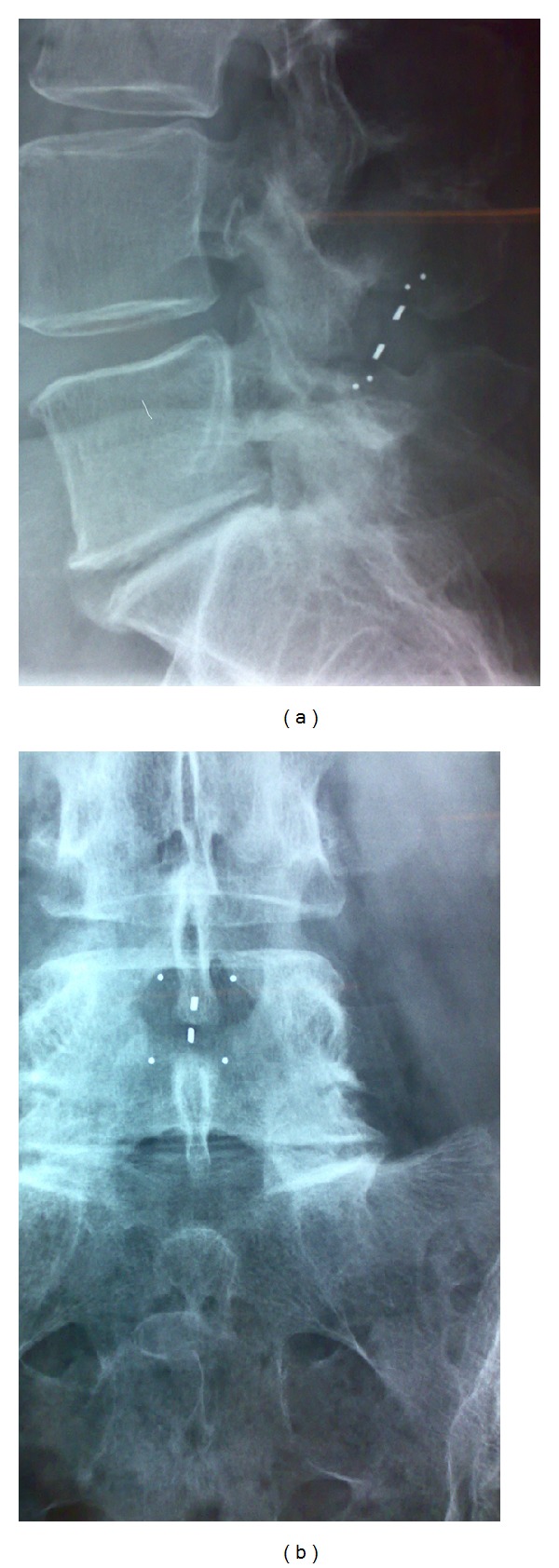
12 months after procedure laterolateral (a) and anteroposterior (b) radiographs are useful to demonstrate the correct position of this L4-L5 implant.

Changes in physical examination data, visual analogue scale (VAS) pain assessments (for leg, buttock/groin, and back), walking condition and distance, use of pain medication, and adverse events (AEs) were also assessed. Immediate postprocedure radiographs were compared with follow-up radiographs by an independent radiologist in order to demonstrate the position of the implant (Figure 2).

Patients were visited monthly in the first three months, and again at 6 and at 12, by a specialized observer (Raul Saggini).

## 3. Results

Baseline data of patients: 90 patients were treated at 1 level and 10 patients (10%) at 2 levels; no patient was treated at 3 levels, even if it was evident that, at entry to the study, 12 patients had several levels of lumbar spinal stenosis. The choice was dictated by the clinical observation data. No particular problems were encountered at L4-L5 level in patients with a prominent iliac crest, and an oblique entry was sufficient to ease implantation. Six weeks after procedure, the mean percentage change in score evaluations indicated a statistically significant improvement from baseline (*P* < 0.005). The statistically significant improvement from baseline was maintained up to 12 months (*P* < 0.005). Statistical analysis was performed with Fisher test, by using The SISA* Binomial* software [40].

(a) Mean VAS improved from 8 to 4, and mean Roland Morris score improved from 16 to 9.

(b) Low back pain, previously present in 62, was abolished in 25, improved in 35, and remained unchanged in 2.

(c) Leg pain, previously present in 47, was abolished in 18, improved in 25, and remained unchanged in 4. The mean score for leg pain decreased from 6 ± 1 at screening to 2 at 7 days, while the mean score for buttock/groin pain decreased from 7 ± 1 at screening to 3 at 7 days. A gradual improvement in back pain was observed: the mean score decreased from 8 ± 1 at screening to 4 ± 1 at 7 days and 3 ± 1 at 6 weeks and maintained thereafter up to 12 months.

(d) NIC, previously present in 84, was abolished in 29, improved in 47, and remained unchanged in 8. Twelve months after procedure, 76% of patients with IPDs implanted were judged by their physician to walk “fluently.” An improvement in walking distance was also observed up to* *12 months after procedure.

(e) Patient satisfaction index (PSI), assessed using the PSQ-18 questionnaire, is as follows: quite satisfied 26 patients, satisfied 66, and nonsatisfied 8. During time PSI did not significantly change.

(f) VAS pain scores for leg, buttock/groin, and back showed that pain decreased significantly (*P* < 0.005) from baseline at all time-points. Immediate improvements were seen in leg and buttock/groin pain after procedure and were maintained up to 12 months.

(g) Medication intake up to 12 months: while pain scores decreased over time, the use of pain medication (including strong pain medication such as opioids) decreased. The number of patients requiring 3 times daily dosing rose 5 days after procedure but subsequently immediately fell. Twelve months after procedure, the percentage of patients requiring pain medication (codeine and paracetamol) was significantly reduced. A high proportion of patients did not require any medication at all (72% of patients versus 25% at baseline).

Data showed a significant improvement from baseline up to 12 months after procedure.

(h) Number of treated levels had no significant impact on changes in pain or Roland Morris scores. Only in the first postop week, the back pain can be considered to be more relevant in double level treatments.

(i) Safety: no serious adverse device effects (SADEs) were reported during this study.

Three minor adverse effects were reported: the first was a marked back pain in one patient due to a huge paravertebral muscles hematoma one day after the procedure; the second and the third were a mild back pain in 2 patients between 2 and 7 days after the procedure.

(j) Device position and removal: final position of IPD in the interspinous space was assessed by X-ray in anteroposterior and laterolateral views, confirming the correct position of IPD throughout the study, with no cases of implant dislocation observed. During the 12-month postprocedure period, 2 patients had their IPD removed after 3 months, due to persistent or recurring symptoms. One device was in L3-L4 and a second in L4-L5. No spinous process fracture or device malpositioning was observed.

## 4. Discussion

Safety and effectiveness of IPD were evaluated for 12 months in DLSS patients with NIC. IPD effectively and quickly alleviated symptoms of spinal stenosis as demonstrated by changes in symptoms severity and physical function scores, VAS pain scores, the use of pain medication, and improvement in walking distance (Table 2).

Data demonstrate that IPD can be beneficial for up to 12 months after implantation in a high proportion of patients, with the majority of patients showing clinically important improvements versus baseline.

Following surgery, an immediate improvement was observed in leg and buttock/groin pain; however, back pain showed a more gradual improvement, which may suggest that patients with classical claudication and leg symptoms as their predominant complaints are the optimal target population for this IPD.

We did not observe a significant long-term effect on the degree of lordosis in our clinical series.

In the literature the most commonly reported SADEs up to 12 months after procedure are back pain, spinal claudication, and spinous process fracture. Spinous process fracture, not observed here, may be considered as potentially risk related specifically to interspinous process decompression, both in terms of the procedure and the device itself [41–44]. Fracture rates, and the means of detecting fractures, vary between studies, making direct comparisons difficult.

During the 12 months after procedure, 2 patients had the IPD removed. In both cases a microdiscectomy was performed, since foraminal stenosis by intraforaminal disc protrusion was persistent and it was considered to be the real cause of nerve dysfunction. The incidence of device removal during this time was low [45–48]. In a recently published systematic review regarding effectiveness of interspinous implant surgery in a total of 563 patients with NIC, 6% of devices required replacement or reoperation [49]. Although it cannot be performed percutaneously, the removal of the IPD is straightforward with minimal risk and leaves other treatment options open (including open decompression surgery, with or without fusion), as fibrotic scar tissue in the spinal canal is not formed following the percutaneous approach. Reoperation does not always require the removal of the IPD.

Analysis on potential predictors for a successful outcome at 12 months in the present study did not reveal new insights to aid patient selection in the future, possibly because the number of patients not benefiting from the procedure was relatively low, and the range of baseline characteristics was wide. More research is needed to define the optimal patient population for this type of procedure. However, the minimally invasive IPD is a viable alternative for selected patients, is quick and simple to implant, and leaves options open for further treatments in the future.

It is known that muscular function is essential for dynamic activity and stability of the spine and that the system is regulated by passive, active, muscular, and neural subsystems. The neural subsystem receives information from the various transducers, determines specific requirements for spinal stability, and causes the active subsystem to achieve the stability goal. Individual muscle tension is measured and adjusted until the required stability is achieved. The requirements for the spinal stability and, therefore, the individual muscle tensions are dependent on dynamic posture and external loads [50]. It is thereby evident that an IPD device which can be positioned without significant muscular lesion and without modification of bony insertions and generation of scarring is ideal in maintaining the natural system in appropriate function.

## 5. Conclusions

Our data indicate that in a period of up to 12-month follow-up, thanks to safety and effectiveness of the HeliFix procedure and device, a minimally invasive option is offered for the relief of NIC complaints in patients with symptomatic DLSS with NIC.

## Figures and Tables

**Table:** (a)

Patients	Male	Female	Mean age
100	42	58	56 y

**Table:** (b)

Symptoms	Yes	No
NIC	84	16
LBP	62	38
LP	47	53
Dist	74	26

NIC: neurogenic intermittent claudication; LBP: low back pain; LP: leg pain; Dist: disability.

**Table:** (c)

Treated levels	Single level	90	58: L4-L5
			32: L3-L4
	Double level	10	9: L3-L4 + L4-L5
			1: L2-L3 + L4-L5

**Table 2:** Summery of patients' outcomes.

	Pretreatment	Posttreatment
Mean VAS	8	4

Mean R-M	16	9

LBP	62	Abolished: 25
		Improved: 32
		Unchanged: 2

LP	47	Abolished: 18
		Improved: 32
		Unchanged: 4

NIC	84	Abolished: 29
		Improved: 37
		Unchanged: 8

PSI	/	Satisfied: 66
		Quite satisfied: 26
		Nonsatisfied: 8

VAS: visual analogic scale; R-M: Roland-Morris disability questionnaire; LBP: low back pain; LP: leg pain; NIC: neurogenic intermittent claudication; PSI: patients satisfaction index.
